# miR-34a expression in human breast cancer is associated with drug resistance

**DOI:** 10.18632/oncotarget.22286

**Published:** 2017-11-06

**Authors:** Zhi-Hua Li, Xueling Weng, Qiu-Yun Xiong, Jian-Hong Tu, An Xiao, Wei Qiu, Yu Gong, Er-Wei Hu, Songyin Huang, Ya-Li Cao

**Affiliations:** ^1^ Department of Breast Surgery, The Third Hospital of Nanchang City, Key Laboratory of Breast Diseases, Nanchang, Jiangxi 330009, P.R. China; ^2^ Guangdong Provincial Key Laboratory of Malignant Tumor Epigenetics and Gene Regulation, Sun Yat-sen Memorial Hospital, Sun Yat-sen University, Guangzhou, Guangdong 510120, P.R. China; ^3^ Department of Pathology, The Third Hospital of Nanchang City, Jiangxi Breast Specialist Hospital, Nanchang, Jiangxi 330009, P.R. China; ^4^ Department of Breast Surgery, Pingxiang People’s Hospital, Pingxiang, Jiangxi 330009, P.R. China

**Keywords:** miR-34a, breast cancer, drug resistance, prognosis

## Abstract

miR-34a is significantly down-regulated in breast cancer tissues and cell lines, which may be correlated with breast cancer multi-drug resistance (MDR). Here, we conducted cell-based experiments and clinical studies in a cohort of 113 breast cancer samples to analyze miR-34a expression and breast cancer MDR. Expression of miR-34a is down-regulated in the multi-drug resistant MDR-MCF-7 cells compared with its parental cells. Patients with miR-34a low expression had poorer overall survival (OS) and disease free survival (DFS) in comparison with those with high expression. Transfecting miR-34a mimics into MDR-MCF-7 breast cancer cells led to partial MDR reversal. Compared with the control group, miR-34a significantly reduced both the mRNA and protein expressions of BCL-2, CCND1 and NOTCH1, but no obvious changes were found in P53 or TOP-2a expression. In breast cancer tissue samples, the expression of miR-34a was related to BCL-2, CCND1 and NOTCH1, but not to HER-2, P53 and TOP-2a. Altogether, our findings suggest that miR-34a is an MDR and prognosis indicator of breast cancer, which may participate in the regulation of drug-resistant breast cancer by targeting BCL-2, CCND1, and NOTCH1.

## INTRODUCTION

Breast cancer is the most common cancer in females worldwide. Although in recent years, the early detection and systemic treatment of breast cancer has made exciting progress, it remains a major cause of cancer related death because of metastasis, relapse, and treatment failure. Multi-drug resistance (MDR) is common in tumor cells and is often the origin of treatment failure. Increasing evidence indicated that the MDR of breast cancer is caused by varying mechanisms. A previous study [1] summarized that the mechanisms breast cancer MDR include the abnormal expression of multidrug-resistance 1 (MDR1), breast cancer resistance protein (BCRP) and other anticancer genes that affect the effectiveness of drugs by decreasing their breast cancer intracellular concentration. MDR mechanisms also include the expression of anti-apoptotic genes such as BCL-2, and P53, which cause cancer cells to resist chemotherapy-mediated apoptosis. In addition, DNA methylation and histone modification have also been proved to be responsible for breast cancer MDR [2–4].

MicroRNAs (miRNAs) are small non-coding RNAs (18–25 nucleotides) which regulate various biological processes by silencing their target genes at the post-transcriptional level. Recent studies have documented that selected miRNAs, such as miR-451, miR-487a, miR-489, and miR-125b play key roles in the chemoresistance of breast cancer [5]. The gene encoding miR-34a is one of the first and well studied miRNAs associated with tumorigenesis, which is located on lp36.23. Li *et al* found that miR-34a is down-regulated in breast cancer cell lines and tissues, compared with normal cell lines and the adjacent non-tumor tissues, respectively [6]. Previous studies have reported that ectopic expression of miR-34a inhibits the growth, invasion and migration of breast cancer cells [7]. It also contributes to drug resistance of breast cancer by targeting a variety of oncogenes. For example, by directly interacting with BCL-2 and CCND1, miR-34a is reported to be associated with docetaxel resistance [8], while by targeting NOTCH 1 and PRKD1, miR-34a modulates chemosensitivity of breast cancer cells to adriamycin [9], and stimulates breast cancer stemness and drug resistance, respectively [10].

MDR is a very complex and multifactorial interaction process. However, the role of miR-34a in the MDR process of breast cancer is still unclear. This study aims to reveal miR-34a expression in breast cancer and its potential role in drug resistance using *in vitro* experiments and clinical studies.

## RESULTS

### Differences of miR-34a expression in drug resistant MCF-7 (MDR-MCF-7) cells and parental MCF-7 cell lines

**Figure 1:**
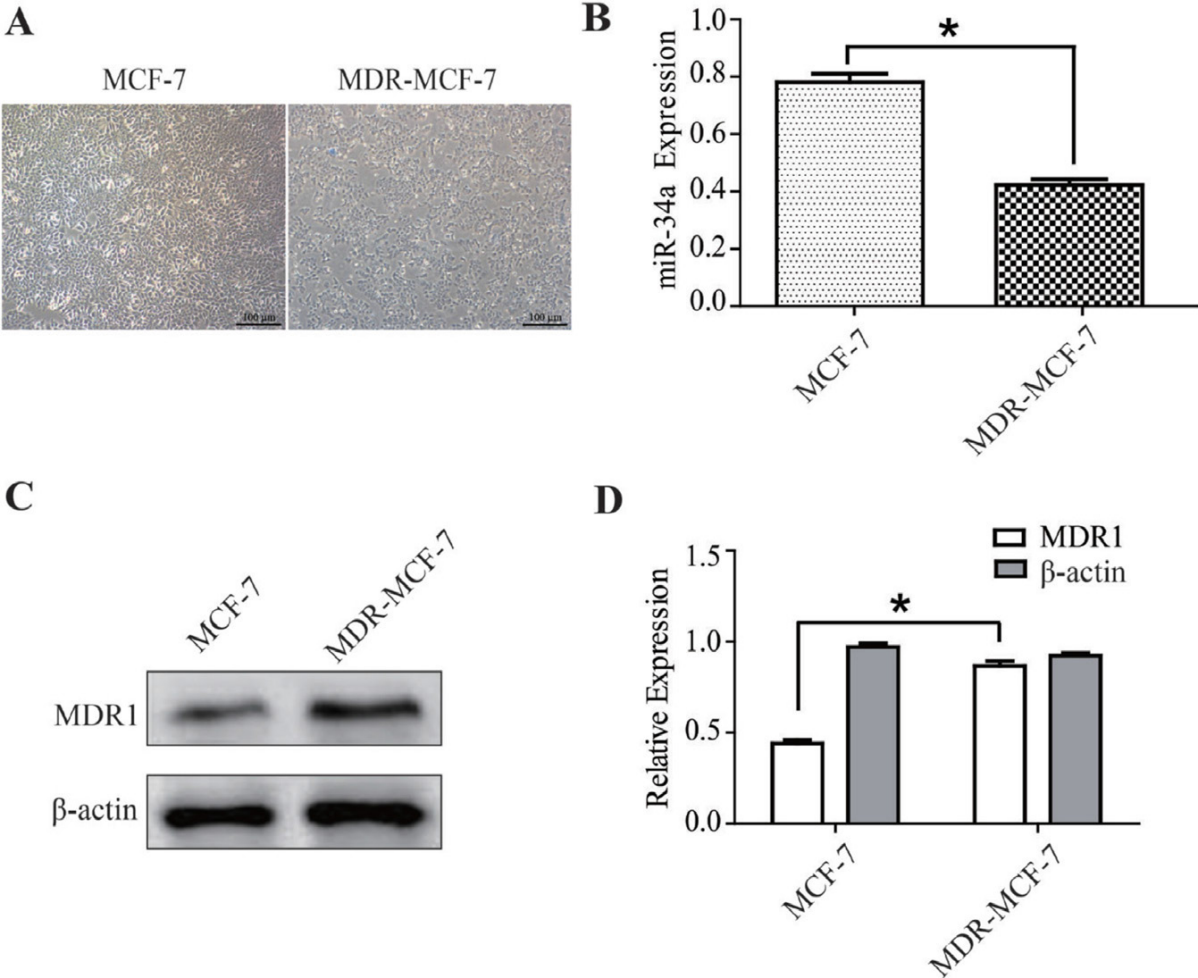
The different expressions of miR-34a in MCF-7 and MDR-MCF-7 cells (**A**) The morphology of MCF-7 and MDR-MCF-7 cells. (**B**) Compared with MCF-7 parental cells, the expression of miR-34a in MDR-MCF-7 multi-drug resistant breast cancer cells was 46% down-regulated. (**C**–**D**) The expression of MDR1 in MDR-MCF-7 cells was significantly higher than in MCF-7 cells. ^*^P < 0.05, as compared with the NC control.

The MDR-MCF-7 cells and parental MCF-7 cells grew well in the logarithmic phase, but the former has more heteromorphism than the latter (Figure 1A). The expression of miR-34a was detected in the two cell lines using qRT-PCR. Expression of miR-34a in MDR-MCF-7 cells was 46% lower than that in MCF-7 cells (*P* < 0.05, Figure 1B). As shown in Figure 1C and 1D, the western-blot result indicated the expression of MDR1 in MDR-MCF-7 cells was nearly twice as high as that in MCF-7 cells.

### Drug sensitivity analysis of MDR-MCF-7 cells transfected with a miR-34a mimic

After transfection of NC-RNA or miR-34a mimics into MDR-MCF-7 cells, MTT assay was employed to detect cell responses to different drug treatments, which include doxorubicin (DOX), cyclophosphamide, docetaxel, and 5-fluorouracil (5-FU). The inhibition concentration curves were plotted to calculate the IC_50_ values. The IC_50_ values in the miR-34a mimic treatment group were lower than those in the NC-RNA mimic group (Table 1), which means that the drug resistance of MDR-MCF-7 cells was partially reversed after transfection with a miR-34a mimic.

**Table 1:** Drug sensitivity of MDR-MCF-7 transfected with NC-RNA and miR-34a mimic

DRUG	MDR-MCF-7 IC50 (umol/L (x ± s)		RI	*t*	*P*
	NC–RNA mimic^*^	miR-34a mimic			
ADM	316.8 ± 43.6	23.2 ± 13.1	13.7	16.67	0.004
DOC	37.5 ± 13.3	6.9 ± 2.4	5.4	4.86	0.04
CTX	9.7 ± 3.7	3.1 ± 1.9	3.1	6.35	0.024
5-FU	6.3 ± 1.4	2.9 ± 1.1	2.2	19.63	0.003

^*^NC-RNA: Negative control RNA.

### Apoptosis and cell cycle distribution of MDR-MCF-7 breast cancer cells transfected with a miR-34a mimic

**Figure 2:**
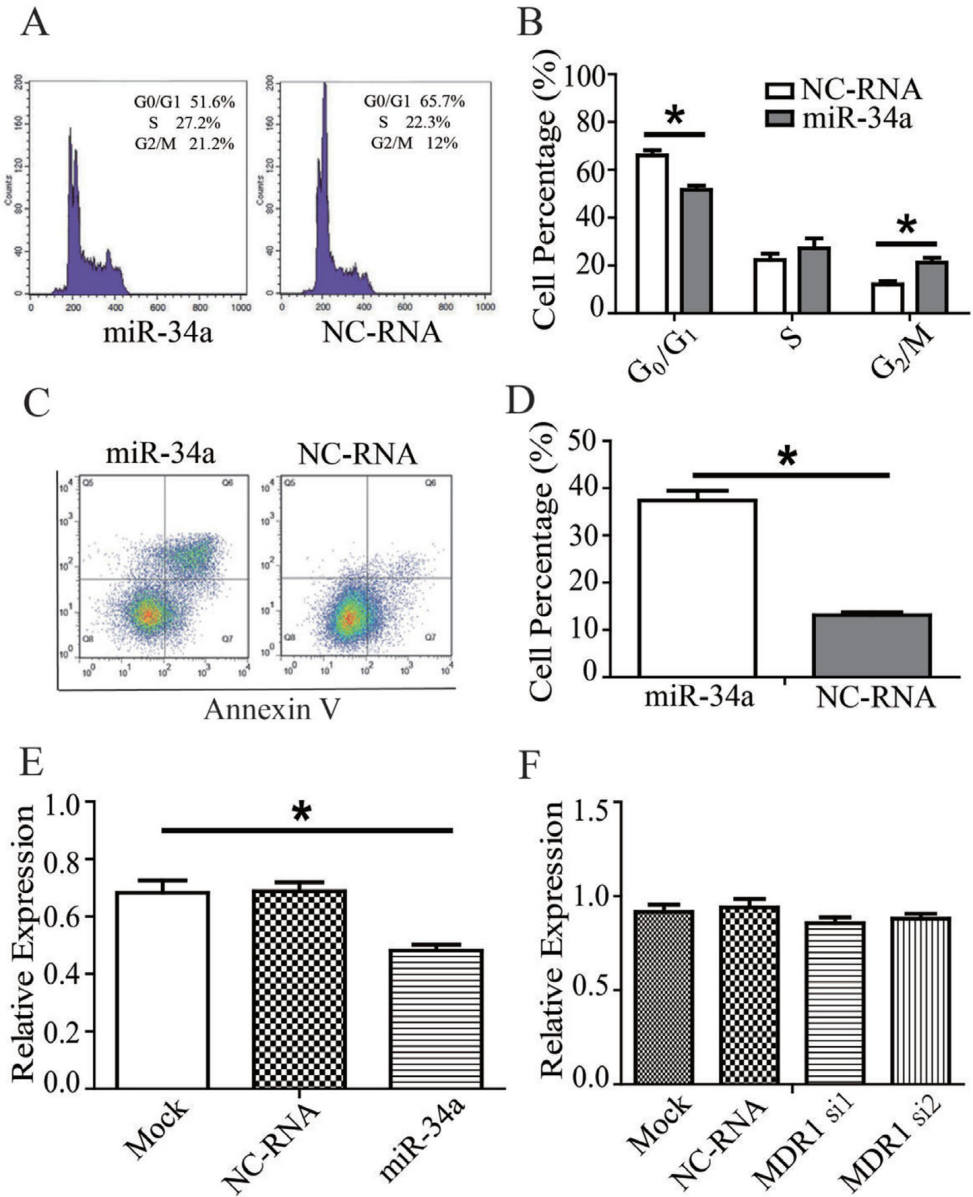
miR-34a effect on the drug-resistance changes of MDR-MCF-7 cells (**A**) miR-34a mimics increase the proportion of cells in mitosis (G2/M phase) and decreased the proportion of MDR-MCF-7 cells in G0/G1 phase compared with NC-RNA mimics. (**B**) miR-34a mimics enhance the apoptotic rate of MDR-MCF-7 cells compared with NC-RNA mimics. (**C**–**D**) The representative flow cytometry figures were also shown. (**E**) miR-34a decrease the expression of MDR1 protein in MDR-MCF-7 compared with NC-RNA. (**F**) The expression of miR-34a was not change in MDR-MCF-7 cells after knocking down MDR1. Bars indicate the mean ± SD from at least three independent experiments. ^*^P < 0.05, as compared with the NC control.

The apoptosis rate of MDR-MCF-7 cells in the miR-34a mimic group was significantly higher than that of the NC-RNA mimic group (*t* = 11.094, *P* = 0.008) according to flow cytometry detection. As for analysis of cell cycle distribution, compared with the NC-RNA mimic treatment, a higher portion of cells transfected with stayed in mitosis (G_2_/M phase) with miR-34a mimic transfection in was higher (*t* = 19.919, *P* = 0.003), and the proportion of cells in G_0_/G_1_ phase was lower (*t* = 20.352, *P* = 0.002). However, no significant difference in the proportion of cells in S phase was detected between the two groups (*t* = 3.395, *P* = 0.077). The data was shown in Figure 2A–2D and Table 2.

**Table 2:** The cell cycle distribute and apoptosis of MDR-MCF-7 transfected with NC-RNA and miR-34a mimic

Groups	cell cycle distribute and apoptosis			
	G0/G1 (%)^**^	S (%)	G2/M (%)^**^	Apoptosis^**^
NC–RNA mimic^*^	51.6 ± 3.1	27.2 ± 7.1	21.2 ± 3.5	13.1 ± 1.1
miR-34a mimic	65.7 ± 4.3	22.3 ± 4.6	12 ± 2.7	36.8 ± 2.6

^*^NC-RNA: Negative control RNA. ^**^P < 0.05.

### MiR-34a decrease the expression of MDR1 protein in MDR-MCF-7

As shown in Figure 2E and Supplementary Figure 1, after miR-34a mimics were transfected into, the expression of MDR1 protein in MDR-MCF-7 cells with miR-34a mimics transfection decreased compared with NC-RNA treatment (*P* < 0.05). But knockdown MDR1 in MDR-MCF-7 cells, significant changes of miR-34a expression were not found (Figure 2F).

### Recovering miR-34a in MDR-MCF-7 breast cancer cells, the expression changes of TOP-2a, P53, BCL-2, CCND1, NOTCH1

**Figure 3:**
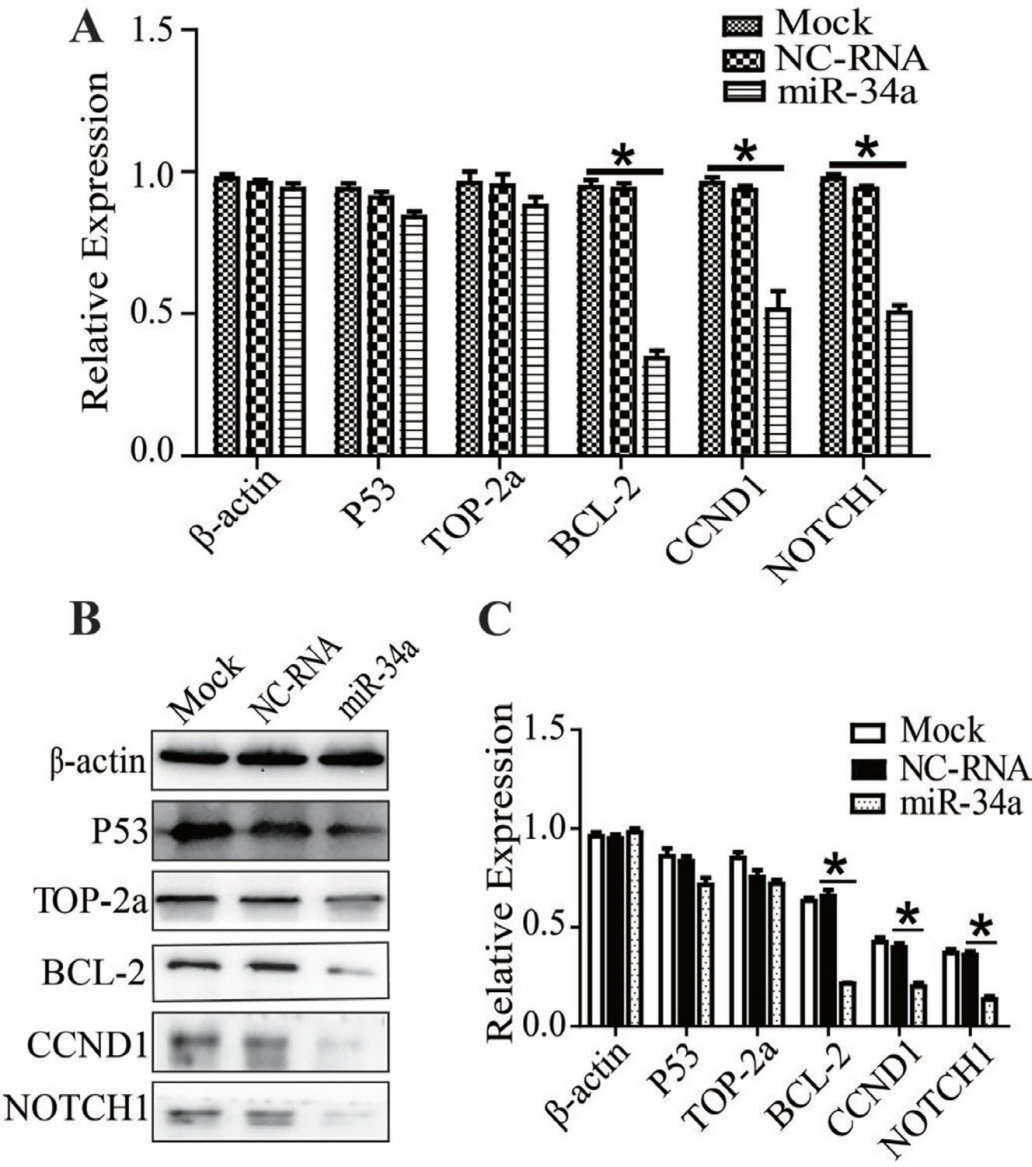
Recovering miR-34a in MDR-MCF-7 cells, the expression of TOP2A, P53, BCL-2, CCND1, NOTCH1 (**A**–**C**) After miR-34a transfection into MDR-MCF-7 breast cancer cells, the expression of BCL-2, CCND1 and NOTCH1 mRNA and protein were both reduced, while the expression of P53 and TOP-2a did not significantly change. ^*^P < 0.05, as compared with the NC control.

After miR-34a or NC-RNA mimics were transfected into MDR-MCF-7 cells, expressions of P53, BCL-2, TOP-2a, CCND1, and NOTCH1 were detected using qRT-PCR or Western-blot. The mRNA expression of P53 and TOP-2a had no significant change compared with NC-RNA mimic group, but the expression of BCL-2, CCND1 and NOTCH1 in the miR-34a mimic group was reduced to 0.43 ± 0.02 (*n* = 3, *P* = 0.03), 0.37 ± 0.01 (*n* = 3, *P =* 0.01) and 0.47 ± 0.01 (*n* = 3, *P* = 0.02), respectively (Figure 3A). Compared with the NC-RNA mimic group, the protein expression levels of BCL-2, CCND1 and NOTCH1 in the miR-34a mimic group were down-regulated, while the expression of P53 and TOP-2a did not change significantly (Figure 3B, 3C). In other words, miR-34a may participate in the regulation of multidrug resistance in breast cancer by down-regulating the expression of BCL-2, CCND1 and NOTCH1.

### miR-34a levels were correlated inversely with drug resistance and poor prognosis of breast cancer patients

**Figure 4:**
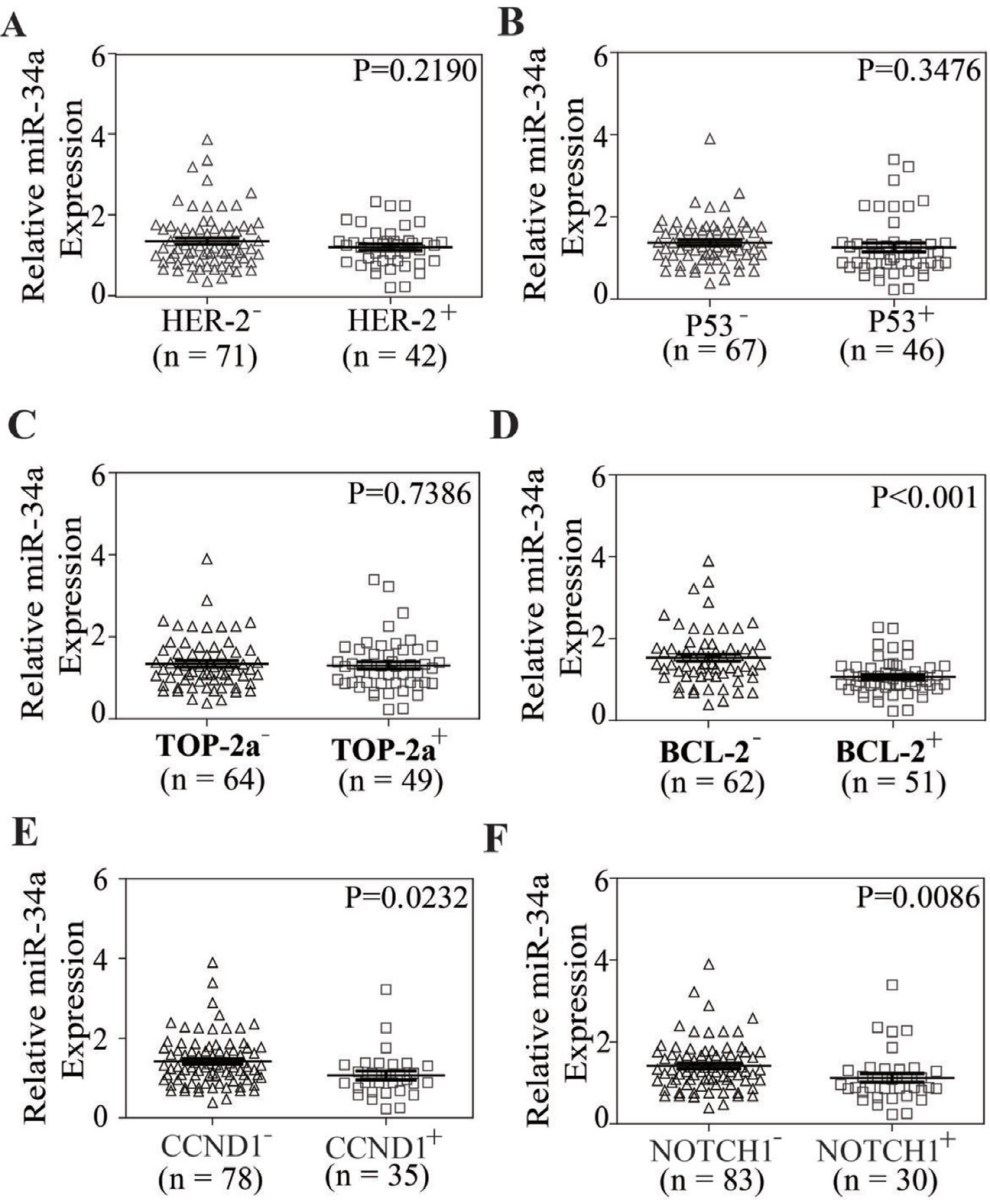
The relationship between miR-34a expression and the expression of HER-2, TOP2A, P53, BCL-2, CCND1, NOTCH1 in breast cancer tissue samples (**A**–**F**) In breast cancer tissue samples, the expression of miR-34a was related to BCL-2, CCND1 and NOTCH1 (*P* < 0.001, *P* = 0.0232, *P* = 0.0086), but not to HER-2, P53 and TOP-2a (*P* = 0.2190, *P* = 0.3476, *P* = 0.7386).

To address whether miR-34a has clinical relevance in breast cancer drug resistance, the expression levels of miR-34a in 113 FFPE tissue samples were examined by qRT-PCR and the expressions of these genes above were analyzed by immunohistochemistry (IHC). The significance was found between miR-34a levels and the expressions of BCL-2, CCND1 and NOTCH1 (*P* < 0.001, *P* = 0.0232, *P* = 0.0086). But the correlations were not found between the expression levels of miR-34a and the expressions of HER-2, TOP-2a and P53 (*P* = 0.2190, *P* = 0.3476, *P* = 0.7386). The analysis data was shown in Figure 4. The correlation between miR-34a expression and clinical-pathological indicators was shown in Supplementary Table 1.

**Figure 5:**
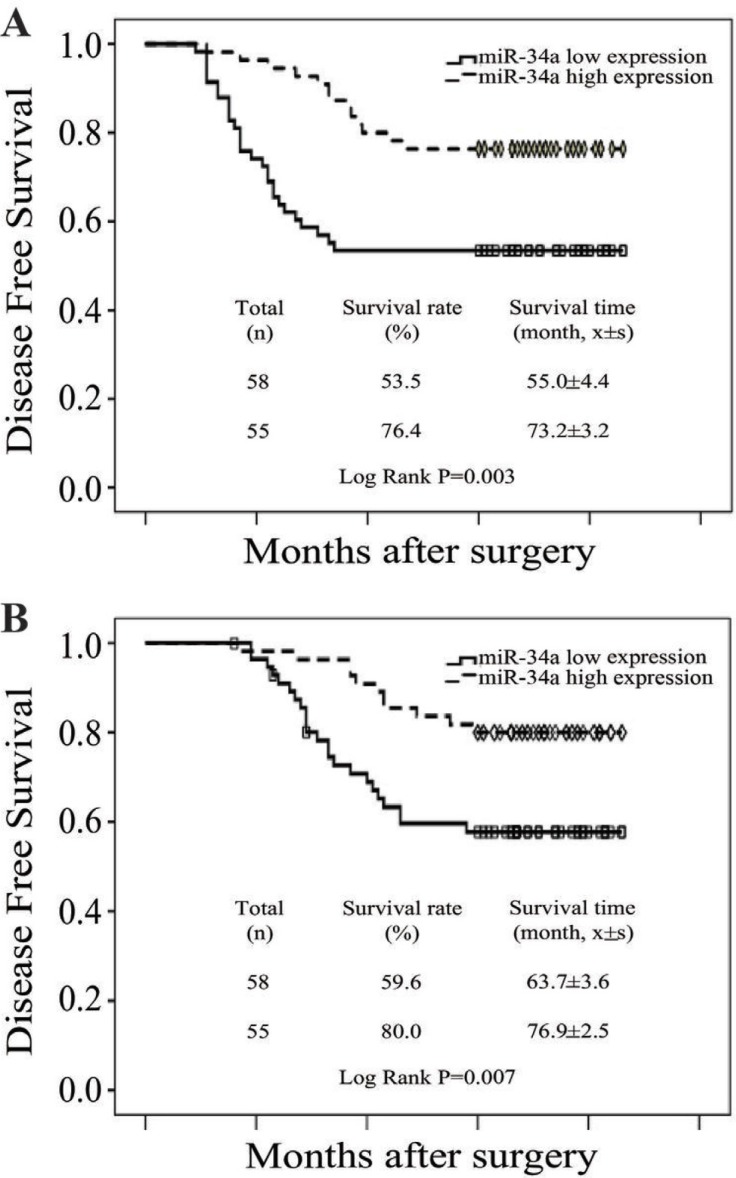
Low level of miR-34a expression was correlated with poor prognosis (**A**) OS curves for 113 patients with low or high miR-34a expression. Kaplan-Meier and log-rank analyses were used. Low levels of the miR-34a were markedly correlated with shorter overall survival. (**B**) DFS curves for 113 patients with low or high miR-34ar expression. Low level of the miR-34a was markedly correlated with shorter disease-free survival. All data are shown as the mean ± SE, ^*^P < 0.05.

To evaluate the prognostic value of miR-34a expression in breast cancer, the Kaplan-Meier survival curve was used to analyze the patient survival outcome. The expression levels of miR-34a in breast cancer were categorized as low or high at the cut-off value of the median. Survival analysis showed that patients with low miR-34a expression had poorer overall survival (OS) and disease free survival (DFS) compared with those with high expression (Log-rank test *P* = 0.003 and 0.007, respectively) (Figure 5A and 5B), revealing that low miR-34a expression indicates poor prognosis for breast cancer patients.

## DISCUSSION

miRNAs are a class of highly conserved, endogenous, small non-coding RNA that are involved in regulation at the post-transcriptional level. The miR-34a gene is located at lp36.23. MiR-34a was firstly identified as a target of P53 and silenced in various human cancers acted as an important tumor suppressor [11, 12]. Increasing evidence has indicated that miR-34a antagonized many different oncogenic processes, such as inhibiting tumor cell differentiation, proliferation, migration, and invasion, and thus increasing apoptosis and cell arrest [13, 14]. Besides, miR-34a downregulation in different types of cancers has been reported to be correlated with cancer multidrug resistance [15]. Recent research has shown that increasing the intracellular expression of miR-34a can significantly increase the sensitivity of cancer cells to cisplatin [16]. Reducing the expression of miR-34a caused resistance in DLD-1 colorectal cancer cells to 5-FU [17], and oxaliplatin induced downregulation of miR-34a and increased drug resistance by activating macroautophagy in CRC cells [18]. Kojima *et al* reported that downregulation or lost expression of miR-34a in PC3PR pancreatic cancer cells led to resistance to taxanes through increasing expression of STIRT1 and BCL-2 [19]. Our study finds that the expression of miR-34a in MDR-MCF-7 cells is lower than that in sensitive parental MCF-7 cells, indicating that miR-34a may be involved in the process of multi-drug resistance in breast cancer.

MDR usually links with treatment failure and poor prognosis of breast cancer patients. It is reported that miR-34a is a diagnostic marker to predict clinical pathological features and outcomes of breast cancer [18, 20]. Our study found that patients with miR-34a low expression had poorer OS and DFS compared to those with high expression, which is consistent with other studies [21, 22], suggesting that low miR-34a expression indicates poor prognosis for breast cancer patients.

Wen *et al* found micellar delivery of miR-34a modulator rubone could reverse chemoresistance and further enhance the therapeutic efficacy of paclitaxel (PTX) in PTX-resistant prostate cancer [23]. Deng *et al* suggested that co-delivery of DOX and miR-34a could achieve synergistic effects on tumor suppression of triple negative breast cancer [24]. Our study discovered that in MDR-MCF-7 cells transfected with miR-34a mimics, the IC_50_ values of DOX, cyclophosphamide, docetaxel and 5-FU decreased to varying degrees, while the apoptosis rate increased and cells were arrested in G_0_/G_1_ phase. After miR-34a mimics were transfected into MDR-MCF-7 breast cancer cells, the expression of MDR1 protein decreased, which implies that miR-34a mimic can partially reverse the drug resistance of MDR-MCF-7 cells, although the underlying molecular mechanism remains unclear. Our result also confirmed that miR-34a may be a therapeutic agent against MDR of breast cancer, which has been a breakthrough in the treatment of HCC patients [25].

**Figure 6:**
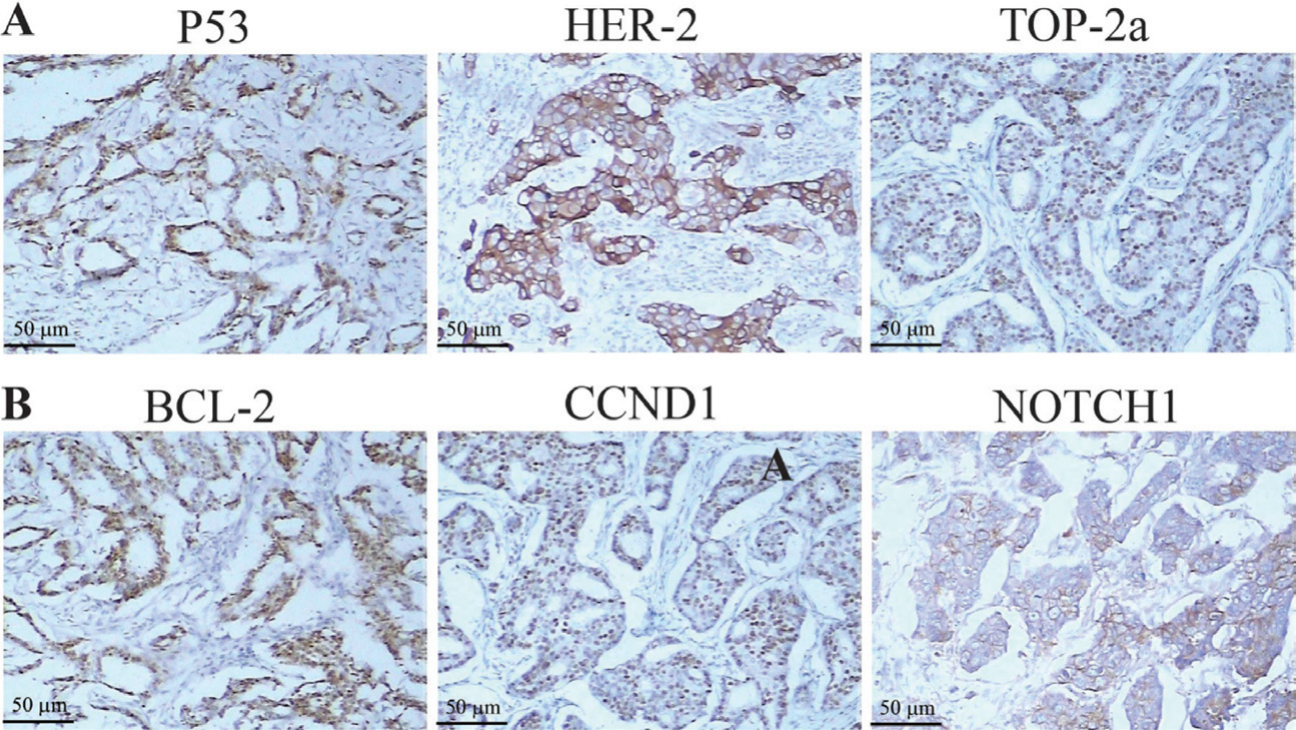
The expression of HER-2, TOP2A, P53, BCL-2, CCND1, NOTCH1 protein were by immunohistostaining (**A**–**B**) Positive cells in breast cancer tissues (SP × 200).

The mechanism of multidrug resistance in breast cancer is very complex. Various studies have demonstrated a series of targeted genes and multiple signaling pathways involved in the regulation of multidrug resistance. Inactivation of P53 as well as activation of PI3K/Akt, RAS/RAF and NF-κB pathways can confer drug resistance to the cells. BCL-2 protein family members leads to the stability of the outer mitochondrial membrane and mitigation of apoptosis, which is another main mechanism facilitating MDR [16]. CCND1, a protein related to cell cycle, was demonstrated to be involved in above EGFR-mediated G1/S transition,which contributed to the multidrug resistance in breast cancer cells [26]. Transcriptional CCND1 expression as a predictor of poor response to neoadjuvant chemotherapy with trastuzumab in HER2-positive/ER-positive breast cancer [27].The NOTCH signaling pathway is a highly conserved signaling pathway. By targeting NOTCH1, miR-34a can regulate the chemosensitivity of breast cancer to DOX [9]. Breast cancer stem cells are considered the source of drug resistance and relapse, thus representing another important mechanism of drug resistance in breast cancer. Studies have shown that NOTCH1 activation is beneficial to maintaining the phenotype of cancer stem cells and promote brain metastasis of breast cancer cells [28]. Additionally, miR-34a can inhibit the proliferation of breast cancer stem cells by down-regulating the NOTCH1 pathway, and miR-34a increases the sensitivity of breast cancer cells to PTX [29]. The TOP-2a gene is located on the 17q21-q22 region and is the target of anthracycline drugs. Changes in intracellular TOP-2a expression and activity form another important mechanism of breast cancer resistance to chemotherapy [30]. The HER-2 gene is located adjacent to the TOP-2a gene and promotes cell proliferation by participating in transmembrane signal transduction in a variety of ways. It increases susceptibility to anthracycline drugs by directly or indirectly enhancing the activity of TOP-2a [31–32]. Meta-analyses suggest that HER-2 and (or) TOP-2a are predictive indicators of chemotherapy resistance in breast cancer [33]. In order to identify miR-34a targets in drug resistance modulation of breast cancer, we investigated the relationship between miR-34a expression and these above-mentioned targeted genes. Our results suggested transfection of miR-34a mimics into MDR-MCF-7 breast cancer cells reduced mRNA and protein expression of BCL-2, CCND1, and NOTCH1 without significantly changing expression of P53 and TOP-2a *in vitro*. In breast cancer tissue samples, the expression of miR-34a was related to BCL-2, CCND1, and NOTCH1, but not to HER-2, P53, and TOP-2a. This indicated that miR-34a is involved in the regulation of drug-resistant breast cancer and may target BCL-2, CCND1, and NOTCH1. These findings are consistent with a bioinformatic analysis (http://www.targetscan.org/) and previous literature reports [8, 29, 34],which were shown in Figure 6. Of course, there are some disagreements on the regulatory effect of P53 on miR-34a. Park *et al* suggested that the reduced level of miR34 is related to the dysregulation of P53 in chemoresistant MDR-MCF-7 cells [35]. However, the expression of P53 did not change significantly when miR-34a was up-regulated .We also found that P53 protein was expressed in a higher level in MDR-MCF7 cells compared with parental MCF-7 cells, which was shown in Supplementary Figure 2 we think there are two reasons for the inconsistent results. Firstly, the positive expression of P53 IHC incompletely represents mutations status of P53 [36]. Secondly, miR-34a might be regulated by other factors which work independent of P53. miR-34a expression can be affected in a significant proportion of breast tumors independent of P53 [37].

In short, miR-34a can serve as an indicator of MDR and prognosis of breast cancer. MiR-34a may participate in the regulation of drug-resistant breast cancer by targeting BCL-2, CCND1, and NOTCH1.

## MATERIALS AND METHODS

### Patients

Patients included in this study were retrospectively selected from the database of breast cancer patients treated between February 2008 and June 2010 at the Third Hospital of Nanchang. All patients are female, which were diagnosed with invasive breast cancer staging II–III, and had perfect follow-up data after treatment. After comprehensively skimming the case archives of every single patient, 113 patients were enrolled in our study. Study protocol was approved by the Ethics Committee of the Third Hospital of Nanchang (NO. 20071208), and all study patients signed consent forms. All patients were treated according to the guidelines for the clinical diagnosis and treatment of breast cancer. Adjuvant chemotherapy used FAC (5-fluorouracil + doxorubicin + cyclophosphamide) or AC-T (doxorubicin and cyclophosphamide sequential paclitaxel). ER-positive tumors were treated with endocrine therapy. If axillary lymph node metastasis were ≥4, tumor size was ≥5 cm or axillary lymph node metastasis was from 1 to 3 with two or more risk factors for recurrence, patients were treated with chest wall + supraclavicular radiotherapy. The median age of patients at diagnosis was 46.3 years (range 31∼73 years). The tumor characteristics are shown in Table 3.

**Table 3:** Clinicopathologic characteristics of patients cohort

Variable			N		%	
menopausal status						
	Pre-menopausal		69		61.1%	
	Post-menopausal		44		38.9%	
Staging						
	I+ IIa		58		51.3%	
	IIb + III		55		48.7%	
Histologic type						
	Invasive ductal carcinoma		78		69%	
	Invasive lobular cancer		18		15.9%	
	Mixed type		7		6.2%	
	Other types		10		8.8%	
ER status						
	ER negative		40		35.4%	
	ER positive		73		64.6%	
PR status						
	PR negative		47		41.6%	
	PR positive		66		58.4%	
HER-2 status						
	HER-2 negative		71		65.5%	
	HER -2 positive		42		34.5	
Surgical types						
	Breast conservation		13		11.5%	
	mastectomy		100		88.5%	
chemotherapy						
	FEC regimen		51		45.1%	
	CET or AC-T regimen		62		54.9%	
Adjuvant radiotherapy						
	YES		53		46.9%	
	No		60		53.1%	

### Cell culture and determination of drug sensitivity

The MCF-7 human breast cancer cell line and the MDR-MCF-7 multi-drug resistant cell line were purchased from the Cell Bank of the Chinese Academy of Sciences (Shanghai, China). The cells were cultured in RPMI 1640 medium (Gibco-BRL, Gaithersburg, MD, USA) supplemented with 10% FBS, 105 U/L penicillin and 100 mg/L streptomycin (Gibco Laboratories, Grand Island, NY, USA). All cells were cultured at 37°C in the presence of 5% CO_2_. MDR-MCF-7 cells were exposed to 0.05 μM doxorubicin for the maintenance of the MDR phenotype, but cultured in drug-free medium for at least one week prior to experimental study. The cell survival rate was detected using MTT (Sigma-Aldrich, Dorset, UK) assay, then concentration inhibition curves were plotted to calculate the IC_50_s of doxorubicin, docetaxel and 5-fluorouracil in MCF-7 and MDR-MCF-7 cells (RI = IC_50_ (resistant cells)/IC_50_ (sensitive cells)). Experiments were repeated three times.

### RNA extraction

Total RNA was extracted from frozen cells using TRIzol^®^ reagent according to the manufacturer’s protocol (Invitrogen, Paisley, UK) and stored at –80°C until use. Archival formalin-fixed paraffin-embedded (FFPE) tissue samples were obtained from the Pathology Department of the Third Hospital of Nanchang. Small punches of FFPE blocks were deparaffinized and treated with proteinase K. RNA was isolated from human FFPE tissue samples using the miRNeasy^®^ FFPE Kit (Qiagen, Hilden, Germany), and RNA quality and quantity were determined using agarose gel electrophoresis and a UV spectrophotometer following manufacturer’s guidelines.

### Reverse transcription and quantitative real-time PCR

For miR-34a analysis, quantitative real-time-PCR (qRT-PCR) was performed using a TaqMan Reverse Transcription Kit and a TaqMan MicroRNA Assay Kit (Applied Biosystems, Foster City, CA, USA) with U6 as the control. For mRNA analysis, qRT-PCR was carried out with SYBR Green Master Mix (Roche Diagnostics GmbH, Mannheim, Germany); β-actin was used as a control. Primer sequences for mature miR-34a, U6 snRNA and miRNA target genes were synthesized by Shanghai Invitrogen Company and are shown in Table 4. All experiments were carried out in triplicate. The data were analyzed according to the comparative Ct method (2^–∆∆Ct^ ).

**Table 4:** Primer sequences for miR-34a, U6 and miRNA target genes

Gene	Foward primer( 5′→ 3′)	Reverse primer( 5′→3′)
miR-34a	GGGTGGCAGTGTCTTAGC	CAGTGCGTGTCGTGGAGT
U6	GTGCTCGCTTCGGCAGCA	CAAAATATGGAACGCTTC
P53	TCTTCCTACAGTACTCCCCT	GCTTGCTTACCTCGCTTAGT
Bcl-2	ACACCCCAATTCTTCCTGCCC	AATCCTCCCCCAGTTCACCC
Top-2a	GCGAGTGTGCTGGTCACTAA	ACAATTGGCCGCTAAACTTG
CCND1	GTCTTCCCGCTGGCCATGAACTAC	GGAAGCGTGTGAGGCGGTAGTAGG
Notch1	CACTGTGGGCGGGTCC	GTTGTATTGGTTCGGCACCAT
β-actin	TCATGAAGTGTGACGTGGACATC	CAGGAGGAGCAATGATCTTGATCT

### Transfection of miR-34a and NC-RNA mimics into the MDR-MCF-7 breast cancer cell line

MDR-MCF-7 cells in the logarithmic phase were collected using trypsin digestion and centrifugation. Then, cells were seeded in 24-well plates and cultured in suspension in complete medium without antibiotics. When cells achieved 30∼50% adherence, miR-34a and NC-RNA mimics (the structural and synthesis information was shown in Figure 6) were transfected into the cells using Lipofectamine 2000 according to the manufacturer’s instructions. After incubating for 6 hours at 37°C under 5% CO_2_ concentration, the transfection efficiency (up to 90%) was observed with a fluorescence inverted phase contrast microscope. The medium was replaced for new cultures, which were then incubated for 24 hours. Changes in cell sensitivity to doxorubicin, docetaxel and 5-fluorouracil were detected using an MTT assay and the cell-cycle distribution and apoptosis changes were detected using flow cytometry.

### Flow cytometry

After transfection with miR-34a and NC-RNA mimics for 48 h, MDR-MCF-7 breast cancer cells were gently scraped, washed twice with chilled PBS (4°C), and suspended at a concentration of l × 10^6^ cells/mL for flow cytometry detection. For cell cycle analysis, target cells were fixed in 75% ethanol and stained with propidium iodide (Sigma Aldrich) supplemented with RNase A (Roche). The Annexin V-APC/7-AAD apoptosis kit (KeyGEN Biotech, Nanjing, China) was used for apoptosis assays. Each set of two wells was analyzed 3 times with a BD Canto II flow cytometer (BD Biosciences, San Jose, CA, USA). Data were analyzed using CellQuest software.

### Western blot

After transfection with miR-34a and NC-RNA mimics for 48 h, MDR-MCF-7 cells were washed 3 times with chilled PBS, and as much residual washing liquid as possible was absorbed. Cells were lysed in RIPA buffer (Sigma). The samples were separated using SDS-PAGE and transferred to PVDF membranes. GAPDH was used as an endogenous control. The proteins of miRNA target genes (P53, BCL-2, TOP-2a, CCND1, NOTCH1) were detected using the Western blot method with ECL development and by analyzing the strength of the strip.

### Immunohistochemistry

**Figure 7:**
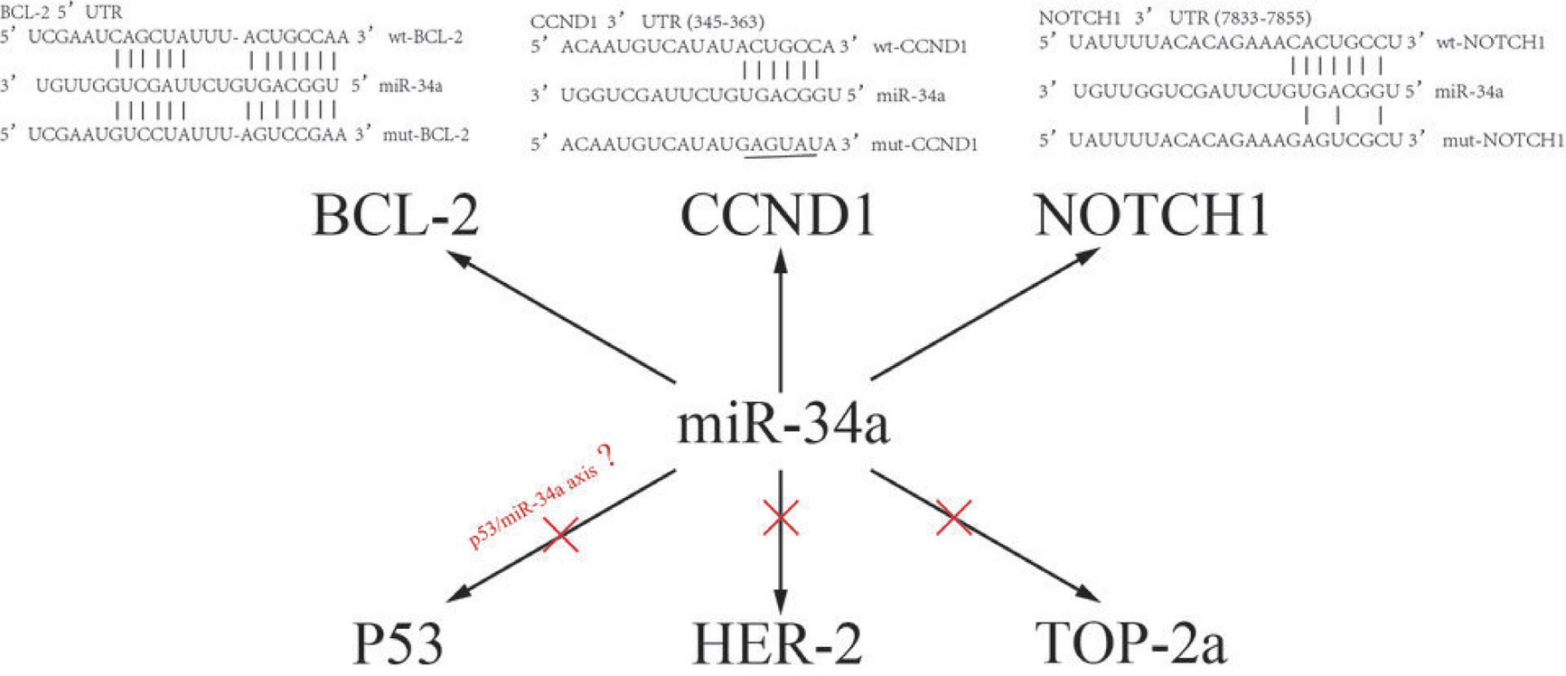
A sketch of miR-34a and their target genes

For IHC, paraffin sections were treated with the following antibodies: TOP2A (Dilution 1:1000; Dako, UK), P53 (Dilution 1:250; Cloning DO7; Dako, UK), BCL-2 (Dilution 1:250; clone 124; Dako, Ely, UK), CCND1 (Dilution 1:1000; SC-8396; Zhongshan Biologicals, Beijing), NOTCH1 (Dilution 1:100; Santa Cruz Biotechnology). Fixed and paraffin-embedded tissue sections were incubated by two independent observers and scored with a scale ranging from 0 to 3 points: 0 score, lack of positive tumor cells; 1 score, cells were stained weakly or <10% positive tumor cells; 2 score, moderate staining or 10–50% positive tumor cells; 3 score, severe staining or >50% positive tumor cells . 0∼1 score was defined as Negative expression and 2∼3 score as Positive expression (Positive image shown in Figure 7).

### Statistical analysis

Statistical tests were carried out using SPSS version 18.0 (SPSS Inc., Chicago, IL, USA) and Graphpad Prism 5. The differences between groups were analyzed using a Student’s *t*-test when only 2 groups or 1-way analysis of variance when more than 2 groups were compared. Differences in frequency were assessed by Chi-square test. The cut-off for the follow-up period was June 30, 2015. DFS and OS curves were calculated using the Kaplan-Meier method and compared by log-rank testing. A *P* < 0.05 was considered statistically significant.

## SUPPLEMENTARY MATERIALS FIGURES AND TABLE



## Abbreviations

MTT: 3-(4,5-dimethylthiazol-2-yl)-2,5-diphenyltetrazolium bromide
MDR: Multi-drug resistance
BCRP: breast cancer resistance protein
FAC: 5-fluorouracil, doxorubicin and cyclophosphamide
CET: cyclophosphamide, epirubicin and taxane
AC-T: cyclophosphamide and epirubicin-taxanes
ER: estrogen receptor
PR: progesterone receptor
HER: human epidermal growth factor receptor
FFPE: formalin-fixed paraffin-embedded
IHC: immunohistochemistry
BC: breast cancer
HCC: hepatocellular carcinoma
DFS: Disease-free survival
OS: Overall survival
TP53: tumor protein p53
