# Supplementary material for: miR-34a expression in human breast cancer is associated with drug resistance
Source: Oncotarget. 2017 Nov 6;8(63):106270–82. doi: 10.18632/oncotarget.22286 (PMC5739732; doi:10.18632/oncotarget.22286)
Supplement: Supplementary file 1 [file oncotarget-08-106270-s001.pdf]

miR-34a expression in human breast cancer is associated with drug resistance

SUPPLEMENTARY MATERIALS

Structural and synthesis information of miR-34a and NC-RNA mimics

miR-34a mimic and negative control mimic (NC-RNA mimic) were synthesized and purified by the GenePharma Company (Shanghai, China). The sequence of miR-34a mimics is 5′-UGGCAGUGUCUUA GCUGGUUGU- 3′. The sequence of NC-RNA mimics 5′-FAM-UUCUCCGAACGUGUCACGUTT-3′.

Design and synthesis of shRNA targeting MDR1

To test whether the regulation of miR-34a in breast cancer cells was directly related to MDR1, we knocked down the MDR1 using shRNA. The sequence of MDR1 hairpin siRNA is 5′-GTATTGACAGCTATTCGAAGAGT GGGCAttcaagaga TGCCCACTCTTCGAATAGCTGTC AATACttt-ttt-3′, which were were designed and synthesized by Forevergen Biosciences (Guangzhou, China). MDR1 shRNAs were transfected into MDR-MCF-7 cells using Lipofectamine 2000 (Invitrogen, CA, USA), according to the manufacturer's instructions. Then the expression of miR-34a was detected in MDR1 shRNA group and control group.

Association between miR-34a expression and clinicopathological characteristics of breast cancer

We detected the expression of miR-34a in 113 cases of breast cancer tissue samples by qRT-PCR. The expression levels of miR-34a in breast cancer were categorized as low or high expression at the cut-off value of the median.

We additionally investigated the association between miR-34a expression and clinicopathological features of breast cancer including menstrual status, histological grade, clinical stage, lymph node metastasis status, ER status and PR status in a univariate χ^2 test. As shown in Supplementary Table 1, no significant differences were observed between miR-34a expression and menstrual status, histological grade, clinical stage, ER status and PR status of breast cancer ($P > 0.05$). However, miR34a expression was significantly associated with lymph node metastasis status ($P = 0.003$).

Supplementary Table 1: Association between miR-34a expression and clinicopathological characteristics of breast cancer

Clinical index	case (n = 113)	miR-34a expression		χ^2	P
		high	low		
menopausal status					
Pre-menopausal	69	33 (60.0%)	36 (62.1%)	0.051	0.822
Post menopausal	44	22 (40.0%)	22 (37.9%)		
Histologic type					
Special type	35	18 (32.7%)	17 (29.3%)	0.154	0.695
Non special type	78	37 (67.3%)	41 (60.7%)		
Axillary lymph node status					
Metastasis	55	19 (34.5%)	36 (62.1%)	8.56	0.003
No	58	36 (65.5%)	22 (37.9%)		
PTNM					
Stage I+II	82	38 (69.1%)	44 (75.9%)	0.650	0.42
Stage III	31	17 (30.9%)	14 (24.1%)		
ER					
negative	40	22 (40.0%)	18 (31.0%)	0.992	0.319
positive	73	33 (60.0%)	40 (69.0%)		
PR					
negative	47	21 (38.2%)	26 (44.8%)	0.513	0.474
positive	66	34 (61.8%)	32 (55.2%)		

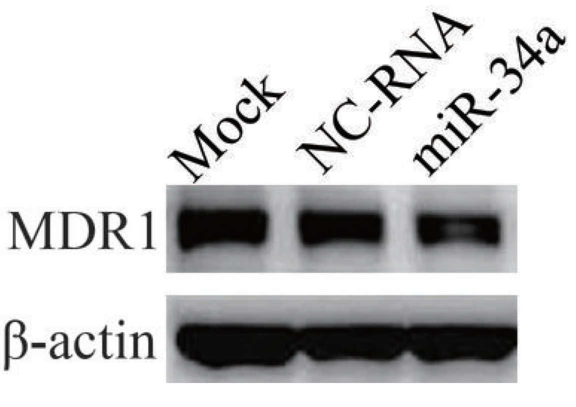

Supplementary Figure 1: miR-34a effect on the drug-resistance changes of MDR-MCF-7 cells. miR-34a decrease the expression of MDR1 protein in MDR-MCF-7 compared with NC-RNA.

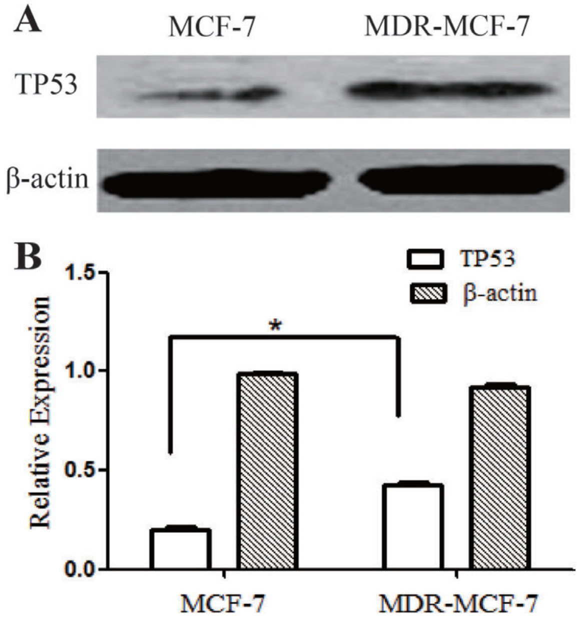

Supplementary Figure 2: TP53 expression in breast cancer cells. (**A**–**B**) TP53 protein was higher expressed in MDR-MCF7 cells compared with its Parental MCF-7 cells.
